# Claude Bernard’s route to the isolation of glycogen: the journey that changed scientific views on the physiological role of the liver and animal metabolism

**DOI:** 10.1007/s00421-025-06080-x

**Published:** 2025-12-19

**Authors:** Jørgen Jensen, Claire Puissant

**Affiliations:** 1 Department of Physical Performance, Norwegian School of Sport Sciences, Ullevål Stadion, P.O. Box 4014, Oslo, 0806 Norway; 2 AgroParisTech, 22 place de l’Agronomie, Palaiseau, 91120 France

**Keywords:** History, François Magendie, Vitalism, Sugar, Glucose, Diabetes, Liver, Homeostasis

## Abstract

Claude Bernard (1813–1878) studied medicine in Paris from 1834 to 1843. During his studies, he attended the physiology lectures at Collège de France provided by François Magendie (1783–1855). Magendie was the world’s leading experimental physiologist, and Bernard became his assistant in 1841. Bernard’s training in vivisection, anatomy and physiology was the foundation for his success as an experimental physiologist. Bernard’s first years of independent research were not successful, but his dexterity in vivisection allowed him to collect pancreatic juice from living dogs in 1848. With fresh pancreatic juice available, he demonstrated the lipolytic action of the fluid. Bernard’s first successful finding had been made. The young chemist Barreswil had established a method for the measurement of glucose in Paris in1845 and became Bernard’s collaborator. In 1848, they reported that the liver contained sugar, whereas other tissues did not. Bernard continued these studies and established the glucogenic function of the liver the same year. This finding transformed the existing view of animal metabolism. Serendipity helped in his next big achievement, enabling him to isolate glycogen. Bernard made always double determinations of sugar but during an experiment in 1855, he was unable to make both the determinations of sugar in the liver on the same day. Bernard had perfused a liver with cold water to remove all glucose but found much glucose in the liver the next day. Bernard acknowledged the surprising result and systematically investigated the formation of glucose in water-perfused livers, finding that the “glucose forming” material was insoluble in alcohol and sensitive to heat. Finally, in 1857, Bernard described a method for isolating glycogen and characterized it as “animal starch”, which could produce sugar. The method Bernard used to isolate glycogen is still used, with some minor modifications. Bernard’s legendary status is unquestionable. He characterised glycogen and introduced the concept of the constancy of the internal environment (*la fixité du milieu intérieur*), which today is known as homeostasis. His fame also survives through the book “An introduction to the study of experimental medicine” published in 1865. The book is still worth reading and should be read in all courses on the theory of science. In this review we discuss the findings that directed Bernard toward the isolation of glycogen.

**Graphical abstract:**

Claude Bernard isolated and characterised glycogen from rabbit liver. Slices of a fresh liver were dropped into boiling water to stop chemical reactions. The liver slices were grounded in a mortar, boiled 30-45 min and filtered. The filtrate was mixed with alcohol and the glycogen precipitated. Bernard did some additional treatment of the glycogen pellet with KOH and acetic acid to remove protein to get pure glycogen. The pure glycogen resembled in all investigations starch and Bernard described it as “animal starch”.

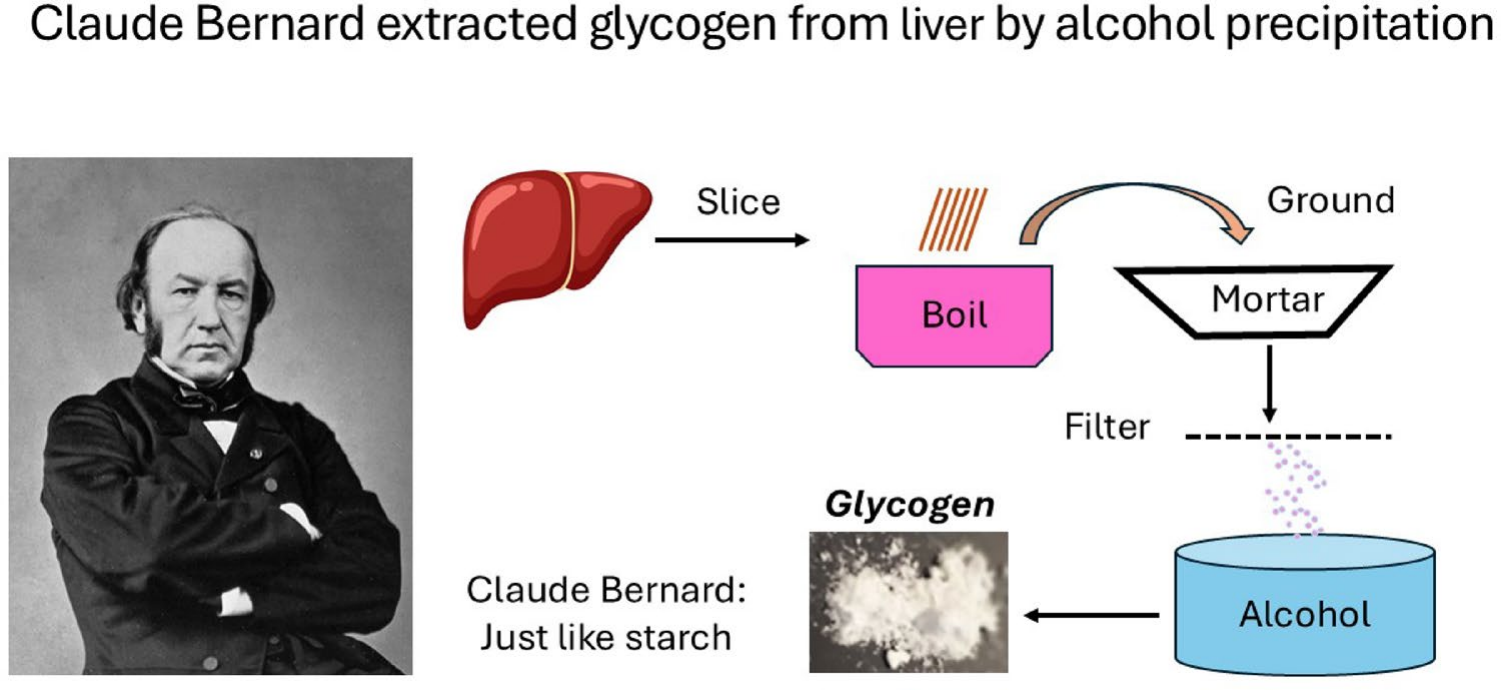

**Supplementary Information:**

The online version contains supplementary material available at 10.1007/s00421-025-06080-x.

## Introduction

Claude Bernard (1813–1878) was a famous French physiologist who contributed to several areas of physiology and published the book “An introduction to the study of experimental medicine”, which is still influential. Both his book and his contributions were highly valued during his life, and Bernard is often called the ‘father of experimental physiology’. Bernard published his work in French and many of his reports were not translated into English.

Paris was the centre for science when Bernard came to the city in 1833. It was not his plan to study medicine, but he was recommended to do so and started his studies in 1834 after he had passed the preparatory exam (baccalaureate). During his medical studies, Bernard was probably more interested in science than medicine, and attended the lectures of François Magendie (1783–1855) at the Collège de France. Magendie was the world-leading experimental physiologist at the time, and Bernard helped Magendie with the preparation of animals from 1839. Still a medical student, Bernard became Magendie’s research assistant in 1841 and was involved in various types of research, which provided vital experience that contributed to his later success.

After finishing his medical studies in 1843, Bernard wanted to study the digestion of nutrients and began to do research in the laboratory of the chemist Théophile-Jules Pelouze (1807–1867). This allowed Bernard to become independent of Magendie and to learn chemistry. Charles-Louis Barreswil (1817–1870), a young chemist working in Pelouze’s laboratory, became an important collaborator for Bernard. Barreswil had established a method for the measurement of sugar (glucose) in 1845 (Barreswil 1845) which allowed them to measure sugar in urine, blood and other tissues, and therefore study the digestion of carbohydrates. In 1848, Bernard and Barreswil reported that sugar was present in large amounts in the liver but not in other tissues under physiological conditions (Bernard and Barreswil 1848). Bernard reported later in 1848 on the glucogenic function of the liver in the paper “On the origin of sugar in the animal body” (Bernard 1848), which changed the view of metabolism in animals.

Bernard continued to study the metabolism of sugar, and serendipity helped him in 1855. In his search for the mechanisms of formation of glucose in the liver, Bernard perfused livers with cold water and was able to remove all sugar from the tissue. Bernard always measured sugar in duplicate, but one day he was unable to make the second measurement and was very surprised when much sugar was found in the liver the next day. Bernard then systematically investigated the reappearance of sugar in a liver that had been washed to remove all glucose. On 24 September 1855, Bernard submitted a paper to the Academy of Science, showing that the liver contained a material that was converted to glucose, and he started to search for this precursor (Bernard 1855b).

Claude Bernard published his famous paper describing the isolation of glycogen in March 1857 (Bernard 1857c). The paper was written in French and difficult to read for most people outside France. We initially intended to make an English translation of the paper. However, with help from the librarian, Thomas Estrier, at Collège de France, we were informed about an English translation published in The Chemist in 1857 (‘On the physiological mechanism of the formation of the sugar in the liver’). This old translation is available in two parts (Bernard 1857a, b) but is unknown to most scientists.

Several papers and books have discussed Claude Bernard’s research and work on glycogen. Young wrote a review for the centenary of the isolation of glycogen (Young 1957) and a more detailed description of Bernard’s theory of the glycogenic function of the liver in 1937 (Young 1937). Chevallier has recently described Bernard’s work on sugar in French (Chevallier 2024). Grmek wrote a historical review of Bernard’s discovery of glycogen from a philosophical perspective (Grmek 1968), and Olmsted and Olmsted described Bernard’s research, including the work leading to discovery of glycogen, in their biography (Olmsted and Olmsted 1952). Holmes carefully studied Claude Bernard’s notebooks from 1843 to 1848 and described his research during this period in “Claude Bernard and Animal Chemistry” (Holmes 1974). Holmes also places Bernard’s research in historical context.

Bernard’s explanations of his discoveries in “An introduction to the study of experimental medicine” (Bernard 1865) and “Lectures on Diabetes and Animal Glycogen metabolism” (*Leçons sur le diabète et la glycogenèse animale*”) (Bernard 1877) do not agree with his notebooks (Grmek 1968; Holmes 1974). The notebooks suggest a complicated and gradual development of the discoveries, whereas Bernard simplified the facts in his book and lectures (Grmek 1968). In this review we describe Claude Bernard’s research on glucose metabolism chronologically, and present the findings that made Bernard search for “a glucose producing material”, and finally isolating and characterising glycogen. The English translation from 1857 of the paper where Bernard reported the isolation of glycogen is included with comments as supplementary material.

## Claude bernard’s early life and education

Claude Bernard was born on July12, 1813, in Saint Julien, a small village in Beaujolais. His father was a wine merchant and landowner, with some hectares of vines, and the family belonged to the rural bourgeoisie (Habert 2022). Bernard was not destined to become a famous scientist and he did not do well at school and failed the baccalaureate in 1831 (Habert 2022). Instead of further studies, he started an apprenticeship in pharmacy near Lyon (Habert 2022; Olmsted and Olmsted 1952). However, Bernard was more interested in theatre and becoming an author and the apprenticeship was terminated.

Young Bernard’s dream was to become a playwright, and he wrote a short play (“La Rose du Rhône”, a vaudeville comedy) and a novel called “Arthur of Bretagne”. At the age of 20, Bernard went to Paris in 1833, with his novel and the ambition to become an author. His attempt to become an author has been described by many, including Kushner’s brief article “Claude Bernard. A failed playwright” (Kushner 2015) and the biography by Olmsted and Olmsted (Olmsted and Olmsted 1952). In brief, the critics turned down Bernard’s novel and suggested he should study medicine since he had some experience from pharmacy.

Bernard stayed in Paris and started to study medicine in 1834 after passing the required exams. He seems not to have been particularly motivated or interested in his medical studies, but passed the examination for an internship in Paris ranked 26th out of 29 students who were successful (Olmsted 1939; Wise 2011). During his studies, Bernard followed Magendie’s lectures on experimental physiology and helped to prepare animals for Magendie’s lectures in 1839, probably as a volunteer. In 1841, Bernard became a *preparateur* for Magendie while still doing his medical studies (Olmsted & Olmsted). Magendie was the leading physiologist in France, who was a pioneer of experimental physiology and did much vivisection (Olmsted 1944). Importantly, Bernard became involved in many aspects of physiology and became an expert in vivisection and anatomy. These skills were extremely important for Bernard’s future successful experiments and interpretation of data. The difference between anatomy and physiology was small in the mid-19th century, and Bernard’s studies on the nervous system were as much anatomy as physiology.

On December 7, 1843, Bernard presented his thesis «The gastric juice and its role in nutrition» (*Du suc gastrique et de son rôle dans le nutrition*) for «*Le Doctorat en Médicine*». The thesis was dedicated to his father, mother and sister (*A mon père*,* a ma mère et a ma sœur*). The thesis was not extensive (34 pages) and was part of his medical studies (Bernard 1843). Bernard found that cane sugar (sucrose) injected into the blood of dogs was secreted in the urine, but no sugar was present in the urine when the cane sugar was treated with gastric juice for some hours before injection.

## Research in Paris in the first half of the 19th century

Paris was the scientific centre in the early 19th century (Ben-David 1970), with scientists like Pierre-Simon Laplace (1749–1827), Georges Cuvier (1769–1832), Jean-Baptiste Biot (1774–1862), François Magendie (1773–1855), Jean Baptiste André Dumas (1800–1884), Jean-Baptiste Boussingault (1802–1887), Joseph Louis Gay-Lussac (1778–1850), Louis Jacques Thenard (1777–1857), Michel Eugène Chevreul (1786–1889), and Théophile-Jules Pelouze (1807–1867). Many foreign researchers studied in Paris; Justus von Liebig worked with Gay-Lussac in 1822–1824 (Shenstone 1901). A huge number of physiologists and physicians visited Claude Bernard and became important in establishing physiology in their home countries. The Danish physiologist Peter Ludvig Panum spent a year with Bernard in 1852–1853 and was later appointed as Professor of physiology at the University of Copenhagen and was crucial in the development of successful physiological research in Denmark (Petersen 1885). Another prominent visiting scholar in Bernard’s laboratory was William Fredrich Kühne (1837–1900), who learned to collect pancreatic juice, and coined the term ‘enzyme’ in 1876 (Kühne 1976). It was also in Paris that Ascanio Sobrero (1812–1888) discovered nitroglycerin in 1847, working in Pelouze’s laboratory. Alfred Nobel (1833–1895) studied in the same laboratory from 1850 to 1852 and discovered later how to use nitroglycerin for the production of dynamite (Kantha 1999). American scientists also visited Paris as part of their training, including Henry Pickering Bowditch (1840–1911), who was important in establishing the physiology department at Boston Medical School (Cannon 1922). Some of Bernard’s lectures were translated to English and used for teaching (Bernard 1867; Bernard 1878b, 1989).

When Bernard was born, in 1813, France was still recovering from the war against Russia in 1812 and he was only two years old when Napoleon was defeated at Waterloo in June 1815. The following year, 1816, was called “The year without summer^1^” and the nutritional status of poorer Parisians was dreadful (Semba 2012). In an attempt to improve their nutritional status, the chemist Jean-Pierre-Joseph d’Arcet (1777–1844) developed an efficient way to extract gelatine (collagen) from bone and offered to prepare bouillon for the poor and the sick in hospitals (Semba 2012). Antoine Cadet de Vaux (1743–1828) also advocated the use of gelatine for the poor (Semba 2012). However, the nutritional value of gelatine was questioned, and a commission of scientists was created in 1831 (Viel and Fournier 2006) and Magendie provided his report to the Gelatine Commission in 1841 (Magendie 1841). Magendie played an important role and his nutritional experiments on animals showed that collagen contained insufficient nutrients (collagen lacks tryptophan). Magendie is considered the first scientist to use rodents in nutritional research (Bing 1931).

François Magendie was an interesting character. According to Olmsted’s bibliography, Magendie was born in Bordeaux on October 6, 1773, where his father was a physician and surgeon (Olmsted 1944). However, the family moved to Paris in 1791, and his father participated in the revolution. François Magendie was eight years old when he came to Paris, but he did not attend school. His father supported Rousseau’s theories on education and François grew up as another “Emile” as described in Rousseau‘s book (Olmsted 1944). However, at the age of ten François wanted to go to school and soon succeeded among his much younger classmates. At 14 years of age, he won the grand prize offered to the best pupils in Paris with his essay “On the knowledge of the rights of man and the constitution” (Olmsted 1944).

In 1795, Magendie’s father arranged for his son to be accepted for instruction by Boyer, a surgeon at Hôpital de la Charité and a private lecturer. François Magendie became Boyer’s favourite student and prosector in anatomy. Magendie soon conducted his own classes in anatomy and dissection, and won a competitive examination for a position at the Faculty of Medicine to teach anatomy in 1807. Finally, in 1808, Magendie completed his clinical examination and defended his thesis. At the age of 24, he had finished his medical studies.

Magendie published his “Précis élémentaire de physiologie“ in 1816 (Magendie 1816). This physiology textbook became very successful and was translated into English in 1822 (titled “A summary of physiology”) with a second edition in 1824 (Magendie 1824) and into German in 1826 (“Lehrbuch der Physiologie”). Magendie’s most famous finding was that the anterior roots of the spinal nerves are responsible for muscle contraction, while the posterior roots are sensory (Olmsted 1944). Magendie showed in 1822 that cutting the anterior roots of the spinal cord prevented muscle contraction but sensory capacity was maintained, while cutting the posterior roots removed sensory aspects but maintained contraction (Jørgensen, 2003). However, the English anatomist Charles Bell had also studied the anatomy of the nervous system, created some reports, and claimed that he was the first to discover the function of the anterior and posterior spinal nerve roots. The Bell-Magendie Law and controversy has been discussed extensively, mostly favouring Magendie as the first to correctly describe the different functions of the anterior and posterior roots of the spinal nerves (Jørgensen, 2003; Olmsted 1944). Magendie was seemingly the perfect mentor for Claude Bernard.

## Research on digestion in Paris when Bernard arrived

A major research topic in the 1830 s was digestion and absorption, and there was much metabolic research in progress in Paris when Bernard undertook his medical studies. The first enzyme was isolated in Paris in 1833 by Anselme Payen (1795–1871) and Jean-François Persoz (1805–1868). They crushed germinating barley in cold water, added alcohol and isolated diastase (α-amylase) as a white precipitate (Payen and Persoz 1833). Diastase became the first enzyme (or ferment) to be purified, and they showed that a dilution of 1:1000 rapidly degraded starch.

Jean Baptiste André Dumas (1800–1884) was a chemist and a central figure in Paris and in the French Academy of Sciences when Bernard studied. It had been known since the late 18th century that plants synthesised energy substrates (sugars) using energy from the sun and that animals got their energy from plants. Dumas proposed in a lecture series in 1841 that plants synthesised energy substrates whereas animals only degraded these substrates (Dumas 1841). This claim created a harsh scientific debate with Justus von Liebig; a dispute that also included claims of originality (Holmes 1974). Von Liebig maintained that animals could make fats from sugars and referred to an old study in which it had been shown that bees could make wax from sugars (von Liebig 1842). The chemist and agricultural scientist Jean-Baptiste Boussingault (1802–1887) supported Dumas and conducted experiments to support his idea. Together they wrote “chemical and physiological balance of organic nature; an essay” in which their theories were described more thoroughly (Dumas & Boussingault, 1844). The arguments von Liebig presented, despite being correct, were not particularly convincing, but Persoz showed in 1843 that geese fed corn accumulated more fat than the fat contained in the corn eaten and his data were accepted (Holmes 1974). This finding was accepted as proof that animals were able to synthesis fat.

Louis Mialhe (1807–1886), a physician and pharmacist, was another successful scientist in Paris and became Bernard’s major rival (Holmes 1974). Mialhe isolated amylase from saliva by ethanol precipitation (animal diastase) in 1845 and showed that the enzyme degraded starch (Mialhe 1845). Furthermore, Mialhe correctly proposed, in 1845, that different ferments were responsible for the degradation of carbohydrates, proteins, and fats (Wisniak 2015a). Bernard criticized Mialhe’s research and considered it too chemical. Holmes allocated a complete chapter in his book to the rivalry between the two successful scientists (Holmes 1974).

Appolinaire Bouchardat (1809–1886), a hygienist at Hotel Dieu, studied digestion and diabetes, and he is sometimes considered the founder of diabetology (Wisniak 2017). Bouchardat understood that fasting reduced glucose in the urine and speculated on malfunction of the pancreas being the principal cause of diabetes (Wisniak 2017). Bouchardat recommended a low-carbohydrate diet for people with diabetes (type 1) and, in collaboration with the chemist Emile Martin, developed a bread containing 80% gluten (protein) and 20% flour (Wisniak 2017).

Bouchardat and the physician Claude Marie Sandras (1802–1856) also conducted research in similar areas to Bernard. In 1845, they collected pancreatic juice and isolated amylase with ethanol precipitation. They showed that the “pancreatic diastase” degraded starch to glucose rapidly and that the powder was sensitive to heat and acid treatment (Bouchardat and Sandras 1845b). Another earlier finding by Bouchardat and Sandras was that “fatty matters” were not degraded in the stomach. In 1842, they had studied dogs at different periods after the intake of food and investigated the contents of the stomach, intestine and chyle, and concluded that “fatty matters” passed into the duodenum in a state of emulsion, as a consequence of the alkali provided by the liver and pancreas (Bouchardat and Sandras 1845a; Holmes 1974).

Magendie also conducted much nutritional research and was the first to conduct feeding studies on rodents (Bing 1931). Bernard was probably involved in these experiments during his medical studies (Dawson 1908). In 1846, Magendie was the first to report that glucose was present in the blood of a healthy dog fed cooked potatoes and some lard for several days, and in the blood of a horse fed exclusively on oats, without glucose appearing in the urine (Magendie 1846; Pearce 2023). The fact that glucose was present under normal physiological conditions contrasted with the common view at the time that glucose was only present in the blood and urine in diabetes.

Diabetes was described in ancient times, when it was recognised that sugar was present in the urine. In early times, diabetes was diagnosed by the sweet taste of the urine (March et al. 2022). The English physician Matthew Dobson (1732–1784) described a sugar-like substance obtained from the urine of a diabetic patient by evaporation in 1776 (Dobson and Fothergill 1776). However, it was not until 1838 that Rees isolated sugar-like material from the blood of a diabetic patient, again by evaporation for purification (March et al. 2022; Rees 1838).

Antoine-Laurent de Lavoisier (1743–1794), considered the ‘father of modern chemistry’, published “Traité élémentaire de chimie” (Elementary Treatise on Chemistry) in 1789. In this text, Lavoisier described the reaction: “grape must = carbonic acid + alcohol” which is considered the first description of a chemical reaction (Holmes 1985). Yeast fermentation of glucose to alcohol and CO_2_ was the accepted method to determine glucose at the time of Bernard (Holmes 1974). Bernard also used yeast fermentation to document that glucose existed in the blood and liver tissue (Holmes 1974).

In the 1840 s, quantification of sugars was difficult, but Karl August Trommer (1806–1879) developed a method to detect sugar using copper sulphate in 1841, which was a great step forward (Trommer 1841). The method built on the fact that sugars (e.g. glucose and fructose) have reducing properties, and can reduce copper (II) in an alkaline solution^2^. The reduction of Cu^2+^ changes the colour of the solution from blue to red, and the colour change is used to determine glucose. In Paris, Barreswil modified this method for measurement of glucose by including tartrate to stabilise the copper ion (Cu^2+^) in 1845, which became critical for Bernard’s research on sugars (Barreswil 1845; Wisniak 2020). Barreswil did not develop the method for accurate measurements of glucose for physiological studies, but for industrial purposes (in the wine industry). An organisation promoting French industry had offered a prize of 3,000 francs for a successful method for the quantitative determination of sugar, and Barreswil participated in the competition with a new method based on a modification of the method described by Trommer (Wisniak 2020). Barreswil was awarded only 1,000 francs because the committee did not consider the method to be definitive (Wisniak 2020). Hermann Christian von Fehling (1812–1885) improved the method further in 1849 (Fehling 1849) and this method was used for more than 50 years to determine glucose in the urine of diabetics. Stanley Benedict (1884–1936) refined Fehling’s method in 1907, but the basic principle of the ability of glucose to reduce copper with characteristic colour change was the same. Benedict’s solution, or its variants, was used for more than 50 years for determining glucose levels in diabetics (Simoni et al. 2002). Table 1 describes the years for key findings on carbohydrate research and Bernard’s contributions.

**Table 1:** Years for key events contributing to Claude bernard’s isolation and description of glycogen

Year	Event
1811	Kirchhoff reported that starch degrades into syrup and sugar
1813	Claude Bernards was born in Saint Julien, a small village in Beaujolais
1833	Payen & Persoz isolated diastase (amylase) form germinating barley
1834	Bernard started medical studies
1839	Bernard assisted Magendie with vivisections at Collège de France
1841	Trommer developed copper method for measurement of sugar
1843	Barreswil modified Trommer’s method for measurement of sugar
1843	Bernard obtained the Medical Degree
1845	Mialhe isolated “animal diastase” from saliva which degraded starch.
1845	Bouchardat and Sandras isolated diastase from pancreatic juice
1846	Magendie showed glucose in blood of dog fed carbohydrate
1848	Bernard reported that pancreatic juice degraded fat (lipolysis)
1848	Bernard and Barrewil showed that sugar was present in the liver
1848	Bernard announced that glucogenic function of the liver
1855	Bernard reported that the liver contained a sugar producing material
1857	Bernard isolated and described glycogen in liver (as animal starch)
1857	Sanson reported glycogen in skeletal muscle
1858	August Kekulé reported the empirical formula for glycogen (C_6_H_10_O_5_)_n_
1878	Claude Bernard died. Awarded state funeral as first scientist in France

Many French scientists did successful research on metabolism in the med 19th century, and their findings were important for Bernard’s isolation and characterisation of glycogen

## Bernard’s early research

After finishing his medical degree, Bernard wanted to do independent research on digestion but failed the examination for a teaching position at the medical school (Conti 2001). Bernard had no intention to practise medicine and applied for several positions, but he did not get an academic position immediately. He started his research career without salary or funding.

Digestion was a major research topic at the time, and Bernard wanted to enter this area. He understood that he needed to add a knowledge of chemistry to his physiological expertise to address the important questions in “animal economy”. Bernard therefore left Magendie’s laboratory to do independent research in the laboratory of Théophile-Jules Pelouze (1807–1867) at the Collège de France. Pelouze was a leading chemist at the time and supported Bernard’s research. Importantly, the young chemist Charles-Louis Barreswil (1817–1870) was the head of Pelouze’s laboratory and became Bernard’s good friend and important collaborator (Wisniak 2020). The method Barreswil had established to measure glucose in 1845 (Barreswil 1845) was crucial to Bernard’s successful studies on sugars.

The notebooks Bernard used to document his research, together with other materials, were catalogued by Mirko Dražen Grmek (1924–2000) between 1961 and 1967 at Collège de France (Holmes 1999). Holmes carefully described the experiments Bernard performed from 1843 to 1848 in his book “Claude Bernard and Animal Chemistry” on the basis of Bernard’s notebooks (Holmes 1974). Grmek published “*Le legs de Claude Bernard*” in 1997 (see the essay review by (Holmes 1999).

Bernard started to study the role of gastric juice in the degradation of starch and protein, and much of this work was done in collaboration with Barreswil in Pelouze’s laboratory. Bernard believed that gastric juice was important for the degradation of carbohydrate and proteins, despite some previous research that had questioned this. Blondlot had used microscopy, found starch granules in the intestine, and concluded in his treatise in 1843 “*that starch is not at all converted to sugar in the stomach as MM Tiedemann and Gmelin had accepted*” (Holmes 1974); p.190. Tiedemann and Gmelin did extensive investigations on digestion and reported starch but no sugar in the duodenum, but sugar appeared in the lower digestive tract suggesting that starch was not degraded in the stomach but in the intestines (Holmes 1975). Tiedemann and Gmelin concluded that sugar was formed in the intestines in 1823 (Holmes 1974).

In the 19th century it was important to develop new theories and claim priority of findings. Bernard probably wanted to develop his own theory on digestion, independent of previous theories, to make a greater impact. Therefore, Bernard studied the digestion of starch and protein in vitro and used the same strategy he had used in his thesis for his medical degree: he treated protein and starch with gastric juice before injecting it into blood vessels and investigating its appearance in urine. Prout had shown that hydrochloride (HCl) was the acid secreted in the stomach in 1823 (Wisniak 2015b), which had been confirmed by Tiedemann and Gmelin. However, Bernard believed that the acid in the stomach was lactic acid, supported by analyses performed with Pelouze (Holmes 1974). Bernard’s studies on digestion early in his career were not notably successful (Holmes 1974).

Another topic Bernard studied was the effect of the diet on urine composition in carnivores and herbivores. It was known that herbivores produced turbid and alkaline urine, but Bernard observed that a rabbit brought in from the market produced clear, acid urine (Arunachalam and Woywodt 2010). Here we see one of Bernard’s valuable traits: take observations seriously. The great English physiologist Michael Forster quoted Bernard as saying to those working with him *‘’Put off your imagination as you take off your overcoat when you enter the laboratory; but put it on again*,* as you do the overcoat*,* when you leave the laboratory. Before the experiment and between whiles let your imagination wrap you round; put it right away from yourself during the experiment itself*,* lest it hinder your observing power*“ (Foster 1899); p. 80. Bernard followed the observation of clear urine in the rabbit from the market, and he showed that rabbits’ urine became clear and acidic during fasting and when fed meat. He concluded that animals fed on their own meat when fasting; they became “carnivores”. This experiment with fasting animals, although not immediately particularly successful, may have been important when he found that the liver produced glucose.

## Bernard’s first successful research

Bernard studied the digestion of carbohydrates/sugars and proteins during the first years of his independent research, but his first success as an independent scientist was when he found that pancreatic juice degraded fat. In a way, the success was founded in Bernard’s dexterity in vivisection, which allowed him make fistulae into the pancreatic ducts of dogs and collect enough fresh pancreatic juice for systematic investigations of its function in the digestion of carbohydrates, proteins and fats. Bernard conducted his first experiments on digestion of fat in December 1846, but he did little research on fat metabolism until 1848. Bernard’s notebooks that reported on the digestion of fat were carefully studied by de Romo, who noted that Bernard gave no explanation of why he included the studies on fat digestion (de Romo 1989).

de Romo described Bernard’s experiments that led to the discovery of the lipolytic action of pancreatic juice in her paper “Tallow and the Time Capsule: Claude Bernard’s Discovery of the Pancreatic Digestion of Fat” (de Romo 1989). Although Bernard’s research on fat is not the topic of this paper, we will briefly summarise the finding that pancreatic juice degraded fat, because it was these experiments that made Bernard famous.

Bernard had an interest in pancreatic juice, and he received a sample from a patient believed to have a pancreatic fistula in November 1847. However, the fluid did not degrade starch to glucose as hypothesised. It had been difficult to acquire pancreatic juice and Bernard had tried several times (Holmes 1974). However, in March 1848 he succeeded in creating a fistula in a large dog and collected gastric juice for several experiments, in which he tested the juice’s ability to digest sugar and nitrogenous compounds, and fat. Bernard’s description of the experiment on fat was described by de Romo: *“Pure pancreatic juice: approximately 1/2 gram*,* to which is added about 5 centigrams [0.05 g] of candle tallow. After 8 hours of continued digestion*,* the liquid is distinctly alkaline. It is a perfectly homogeneous*,* whitish emulsion*,* no liquid rises to the top even on exposure to cold or heat*,* the emulsion is fine as milk and contains no grains. Therefore*,* there has been a distinct reaction of the pancreatic juice on the fat. The liquid emulsion remained very alkaline. More experiments on this subject must be done…*” (de Romo 1989).

de Romo noted that Bernard studied the role of pancreatic juice in the degradation of sugars and proteins, and it was surprising that he had no other fat available than candle tallow. Fortunately for Bernard, the triglycerides in candle tallow are degraded just like dietary triglycerides. On Monday, April 3, 1848, Bernard delivered a sealed package to the Académie des Sciences containing the description of his experiments, entitled “Properties of Pancreatic Juice”. This would allow Bernard to claim priority on the idea that pancreatic juice degraded fat, but the package was not opened until 100 years after Bernard’s death (de Romo 1989).

Bernard now focused his research on pancreatic juice and stated that it contained “organic matter” very similar to albumin. Today we know that the proteins Bernard observed were enzymes. However, the fact that enzymes/ferments were proteins was not known at the time and to use de Romo’s translation of Bernard’s notes: *“It is very difficult to determine these characteristics with any greater accuracy because of the virtual impossibility*,* in the present state of science*,* of clearly separating these proteinaceous substances one from another”* (de Romo 1989); p. 266.

Bernard tested the role of pancreatic juice on dietary fats such as lard, butter and oil in April 1848. When Bernard conducted experiments, he was open-minded and investigated various options. An important finding was that pancreatic juice produced an acid solution. Chevreul had found that fats (triacylglycerols) were composed of fatty acids and glycerol (Carpenter 1998; Leray 2023) and Bernard’s creative mind led him to investigate whether fatty acids were produced by the actions of pancreatic juice on fats. On April 16, Barreswil analysed the emulsion created by fat and pancreatic juice and found free fatty acids (de Romo 1989). Bernard now knew that pancreatic juice produced fatty acids from fats (Holmes 1974), and concluded therefore that the pancreatic juice had a lipolytic action and degraded fat to fatty acids and glycerol.

Bernard provided also anatomical support for the lipolytic action of pancreatic juice. He had observed that the pancreatic duct entered the intestine at different places in dogs and rabbits; in dogs the pancreatic duct enters the duodenum next to the pylorus whereas the pancreatic duct enters the intestine 30–40 cm from the pylorus in rabbits. Bernard fed a rabbit fat and killed it 7 h later, observing white chyle only in the lymphatic vessels 2–3 inches below the pancreatic duct (de Romo 1989). This supported the supposition that pancreatic juice was responsible for the degradation of fat.

Bernard made a presentation titled “On the use of pancreatic juice” (*Sur les usages du suc pancréatiques*) to the Philomatic Society on Saturday April 29, 1848, in which he described the lipolytic action of pancreatic juice (de Romo 1989). Bernard’s work on the pancreas was awarded the 1848 Prix de Physiologie Expérimentale of the Académie des Sciences and the red ribbon of the Légion d’Honneur the following year. Apart from the publications from his lectures, Bernard devoted only two other articles to the action of pancreatic juice. His first paper, read on February 1849 before the newly founded Société de Biologie, was entitled “Research on the uses of pancreatic juice in digestion” (*Recherches sur les usages du suc pancréatique dans la digestion*). In 1849, Bernard made a more detailed report in a short book (Bernard 1849). Interested readers are recommended to read the complete story in de Romo’s excellent historical review (de Romo 1989).

## Further studies on glucose digestion and utilisation

During his studies of the lipolytic action of pancreatic juices, Bernard also observed that pancreatic juice degraded starch “extremely fast”. This finding challenged Bernard’s previous theory of the key role of gastric juice in digestion of starch. Bernard’s strength, in addition to his expertise in anatomy and dexterity in vivisection, was his physiological theorising about his data and creating ideas. Bernard believed in experimentation and is famous for the quote: ”*When we meet a fact which contradicts a prevailing theory*,* we must accept the fact and abandon the theory*,* even when the theory is supported by great names and generally accepted*” (Bernard 1865); p.164. Bernard followed his rule – at least sometimes. Although his data were not always consistent, he continued to generate new ideas and perform experiments to understand the physiology of digestion.

Bernard now accepted that carbohydrate was digested and taken up by the intestine, and his new question was: where was the sugar destroyed? Lavoisier had shown in the late 18th century that oxygen was used by animals and compared respiration with combustion (Holmes 1985). Therefore, Lavoisier and most physiologists believed initially that the oxygen was used in the lungs. Sugar was called *respiratory nutrient*, and Bernard believed that the glucose was destroyed in the lungs (Bernard 1865). He wanted to test this idea.

Apparently out of the blue, Bernard ground up rabbit lung tissue in some ordinary water and incubated the tissue with grape sugar overnight in a water bath at 40–45 °C. He found that the sugar had disappeared the next day (Holmes 1974); p. 418. On subsequent days, similar experiments were conducted with other tissues as counter-experiments. These experiments were rather inconclusive and the fact that other tissues also degraded glucose was incompatible with the theory Bernard tested. Retrospectively, an interesting experiment was one in which Bernard found much glucose in liver tissue after incubation overnight, but it would take another 6–7 years before he understood that the liver produced glucose. The strategy with tissue incubation in water seems rather simple today, and Bernard understood that this strategy did not work. However, 50 years later the incubation of ground tissue became an important methodology (Barnett and Lichtenthaler 2001) and today homogenisation of tissues for analyses are widely used (Lai et al. 2007; Whitehead et al. 2000).

Bernard now returned to his old strategy of injecting sugar into animals. This time his approach was more sophisticated, and he designed an experiment in which blood sugar was measured after the blood had passed the lungs. He injected glucose into the right jugular vein and sampled blood from the right carotid artery after the blood had passed through the lungs. Bernard’s initial idea was that the glucose was rapidly degraded in the body, but to his surprise, much glucose was observed in the blood after it had passed through the lungs (Holmes 1974). The measurement of glucose was based on the change in colour of cupric oxide, and Bernard probably compared colours by eye. Bernard initially noted glucose concentrations as “200”, “100” or “10” (Holmes 1974), probably based on a standard curve he had prepared. However, Bernard understood that the method for measurement of sugar was not accurate and started to describe sugar in blood as “huge amount of sugar”, “trace reduction”, or “no sugar” (Holmes 1974). Fulton stated that the method Bernard used for measurement of glucose was not sensitive below 80–100 mg/dl (~ 5 mM) (Fulton 1932), which is a typical concentration in the blood of dogs. Bernard understood that his measurement of glucose in blood was not accurate enough to answer his research question and that he needed another strategy. Today arterio-venous tissues are used to study glucose uptake in specific tissues (Hingst et al. 2022) and Bernard was probably the first to try.

## A new direction of research: sugar in the liver

Bernard often addressed several questions in experiments with a single animal. In early July 1848, he performed experiment on a dog that was fed only bones on the day before. Bernard injected potassium prussiate (salt of cyanide) into a vein and killed the dog to search for prussiate in various body fluids but only found it in the urine. Importantly, he also measured sugar, which was present in blood serum (and in chyle and the vitreous humor of the eye), but not in the stomach and intestine. Bernard noted his speculation about where the sugar came from, and what caused the reduction of Barreswil’s reagent. Bernard concluded in his note book: “*All of that is very curious and needs to be investigated by new experiments*”. (Holmes 1974); p.420.

The experiment that proved crucial was conducted between August 10 and 17, 1848, but the exact date is not noted (Grmek 1968). A dog was fed only meat for eight days before it was killed by sectioning its brainstem to allow collection of blood from various sites. Bernard found “enormous reduction” of Barreswil’s reagent in the serum from the portal vein and “very clear reduction” in the blood from the heart (Holmes 1974). Bernard also investigated the sugar content in the serum by yeast fermentation and found that the yeast “fermented with an unbelievable activity”. In 1848, the accepted method to document sugar was by yeast fermentation, and the copper sulphate method was not completely accepted.

Grmek considered this experiment the turning point in Bernard’s research (Grmek 1968). Bernard had started out searching for the place where glucose was destroyed, but understood that he was unable to do that. Importantly, he understood immediately the significance of the surprising finding of glucose in the portal vein and switched his research to attempting to determine how glucose was produced. Grmek published the exact text from Bernard’s notebook from that experiment (two printed pages in French) in his article “First steps in Claude Bernard’s discovery of the glycogenic function of the liver” allowing readers to understand Bernard’s surprise at the results (Grmek 1968). At the end Bernard wrote: *This experiment is very unique. It’s impossible to understand anything. Sugar would form in the portal vein. By what organ*,* by what mechanism? …. So what is the organ that would form this sugar or this reducing material?*

The direction of Bernard’s research changed to the search for the site of glucose production and he entitled his next experiment “Digestion of fat – formation of sugar at its expense” (Holmes 1974). A dog was fed meat scraps for some time and given a copious meal of meat and lard in the morning before being killed in the early afternoon (August 22). Bernard again collected blood after anatomical investigations of fat absorption. Bernard had speculated on where the glucose in the portal vein was produced, and this time he collected blood from both the furthest branch of the portal vein and the part closest to the liver. Bernard had to press the liver to obtain a blood sample close to the liver and found “enormous reduction” with Barreswil’s solution. In the blood from the furthest branch Bernard only found “trace reduction”. He then searched for glucose in the surrounding tissues and found “the tissue of the liver contained it enormously” whereas the other tissues close to portal vein (spleen and mesenteric ganglion) did not show sugar distinctly (Holmes 1974). Immediately, Bernard purchased beef and calf livers from the butcher and detected great amounts of sugar with Barreswil’s reagent and by fermentation.

The next morning, Bernard went to the Hospital de Charité and obtained liver samples from three deceased patients and found great amounts of sugar in one of the samples. Bernard’s experiments were not consistent. He tried to extract sugar with alcohol and found a reaction with Barreswil’s reagent but not with fermentation. Bernard continued to measure sugar in livers from different animals under various feeding regimes and in samples from human cadavers, but the results were very variable: sometimes he found large amounts of sugar, whereas sugar was not found in other samples, particularly when the liver was fresh. Holmes cited Bernard’s notes: “*Often I believed that there was not any sugar in the livers when I examined them fresh – upon what does that depend? It is undoubtable the liver tissue that is too hard and does not easily yield sugar*” (Holmes 1974). Again, Bernard was close to his big finding of the “glucogenic matter” (glycogen) that produces glucose after death (see discussion below). In this brief period Bernard investigated sugar in livers from dead patients, cold-blooded animals and calves, but did not find sugar in all livers.

On August 25, Bernard and Barreswil reported to the Academy of Science that sugar was present in the liver (Bernard and Barreswil 1848). The report in Compte Rendu is short and not even a full page. At the meeting Bernard and Barreswil presented a sample of alcohol from fermentation of sugar from the liver to the members of the Academy (Bernard and Barreswil 1848). They explained that they would have preferred to provide crystalised sugar, but they had only managed to obtain a syrup/molasses solution loaded with salts, which would not crystalise. In addition, they stated that sugar could be extracted from the liver but not from any other organs (Bernard and Barreswil 1848). Bernard and Barreswil had also found sugar “*in animals completely deprived of sugary or starchy matter*,* and submitted for a long time to an exclusive diet of meat. From this we conclude that the existence of sugar in the liver is a physiological fact completely independent of the nature of the diet*” (Bernard and Barreswil 1848). Bernard did not consider the results sufficient for a full report, but a few days later (August 28) Bernard delivered a sealed letter to the Academy of Science claiming priority on the finding of glucose in the liver (Holmes 1974).

The experiments in September were not very successful. One question Bernard addressed was whether sugar remained present during fasting. In one experiment, a dog was fasted for seven days, became very thin and got pneumonia. Bernard did not find sugar in the blood or the liver. In another dog that had been fasted, he found sugar in blood samples taken after 3 days, but neither in the blood nor the liver when it was killed after 6 days of fasting. We now know that glycogen depletion occurs in the liver after 24 hours’ fasting in both animals and humans (Kolnes et al. 2015; Nilsson and Hultman 1973). Another problem Bernard faced was simply the analyses with Barreswil’s reagent. In one experiment, Bernard used two different preparations of Barreswil’s reagent and found sugar using one of them but not the other (Holmes 1974). Bernard understood the problems and considered various reasons for the variable results, including the way of killing the animals and the duration of fasting.

In early October the results became more reliable, and Bernard consistently found sugar in both blood and the liver (Holmes 1974). He also started to investigate animals that had only been fasted for a few days, and in some experiments fed dogs only meat (i.e., a carbohydrate-free diet). In October, Bernard found sugar in a dog fasted for six days. Another dog fasted for eight days and fed fat only for seven days contained “enormous” amounts of sugar in both the liver and blood. Bernard repeated the experiment with nearly identical results. In these experiments, Bernard killed the dog with a blow to the head. Killing animals this way activates the sympathetic nervous system, which influenced the results of Bernard’s experiments and helped him in his findings (this is discussed later). Bernard also found sugar in the liver of a duck that had been fed washed egg white for 15 days. From these experiments Bernard concluded that “formation of sugar in the liver is independent of aliments”. In this busy period, Bernard kept only brief records of his experiments and Holmes concluded that it was difficult to trace the thoughts that led to his conclusions (Holmes 1974).

## The glucogenic function of the liver

Bernard’s strength laid in both his dexterity in vivisection and his creativity in developing theories from inconsistent data (both his own and others). His mentor Magendie may have contributed to both talents. Magendie was undoubtedly the leading experimental physiologist of the time and an expert vivisectionist, which was important for Bernard’s training. However, Magendie did not theorise much about his data and he is cited as saying: *“Everyone compares himself*,* in his own sphere*,* with someone greater than himself: with Archimedes*,* Michelangelo*,* Newton*,* Galileo*,* Descartes*,* and so on. Louis XIV compared himself with the sun. As for myself*,* I am much more modest. I compare myself to a ragpicker. With my spiked stick in my hands and my basket on my back*,* I walk through the streets of science and pick up whatever I find.”* (Tarshis 1968); p. 71–72. Magendie fought Bichat’s vitalism, and his manifesto from 1809 is considered by some as the start of the new physiology, building on experimental fact only (Schiller 1968). Still, Magendie accepted the idea of some form of vital force, as most physiologists did at that time (Olmsted 1944). Magendie probably considered it too early to develop theories about physiological phenomena, because the data were insufficient. Bernard was different and Magendie’s opposition to theorising may have inspired Bernard to do so. Bernard understood that progress in science required theories that were tested by experiments (Bernard 1865).

On October 21, 1848, Bernard presented the results of his experiments to the Société de Biologie and described the glucogenic function of the liver. There is an English translation of this paper, titled “*On the origin of sugar in the animal economy*” (Bernard 1848). Holmes also carefully discusses this presentation, which is considered a milestone in Bernard’s career and a historic moment in physiology (Holmes 1974). For the presentation, Bernard had selected four series of experiments, in a different order from that in which they were conducted.

In the first set of experiments (a rabbit and three dogs), Bernard reported that sugar was always present in dogs or rabbits whether fed carrots, starch, meat, or fasted for two days. Bernard claimed that he had reproduced these experiments “*a great number of times with similar results*” (Holmes 1974).

In the second series of experiments, Bernard reported that dogs fed meat or fasted had much sugar in the portal vein, less in blood from the heart and nothing in the stomach or lymphatic system. Bernard again claimed that these experiments were repeated many times with similar results and led him to conclude that an organ in the abdomen produced sugar.

In the third series of experiments, Bernard reported data from an experiment where he had ligatured the portal vein and only found sugar in the part closest to the liver. He then analysed pieces of the organs close to the portal vein and found sugar in the liver, but not in the other organs.

The fourth series of experiments were conducted to understand where the sugar came from and Bernard argued that glucose was formed in animals, and concluded that “*sugar forms in the liver*,* and … this organ is at the same time the seat and the source of sugar material in animals*” (Bernard 1848).

## Nervous control of hepatic glucose secretion and research on other areas

Bernard’s next question was: How was sugar secretion from the liver regulated? He had previously investigated the neural regulation of saliva secretion, and he believed that glucose secretion from the liver also was under neural control. He investigated this possibility by stimulating/irritating the nervous system and by cutting nerves (Olmsted and Olmsted 1952; Young 1937). In April 1849, Bernard reported that wounding the floor of the fourth ventricle caused sugar to be released in the urine in both dogs and rabbits (Olmsted and Olmsted 1952); p. 66. Bernard called this artificial diabetes (Holmes 1974). In an attempt to understand the process better, Bernard cut the vagus nerve, but the secretion of glucose remained. Bernard did successful research on the autonomic nervous system, but although he pursued his studies on nervous regulation of sugar secretion from the liver, the outcomes were not fruitful.

Bernard’s research portfolio was broad and he investigated the action of curare between 1849 and 1852 (Olmsted and Olmsted 1952). Pelouze had received curare from a Frenchman living in Brazil, who had bought the poison and some poisoned arrows from the local Indians (Tarshis 1968). In these experiments, curare was inserted under the skin of a frog and when the effect was evident, the skin was removed, and the motor nerve exposed. Bernard and Pelouze reported that curare prevented muscle contraction mediated by nerves, but the muscle still contracted with direct stimulation (Olmsted and Olmsted 1952). These data showed that it was the contact between the nervous system and the muscle that was affected, and we now know that curare blocks the acetylcholine receptors at the neuromuscular junction and prevents the nerve impulses from activating muscle contraction. The work on curare also illustrates Bernard’s brilliance as an experimental physiologist, although the mechanism of action Bernard presented was not completely correct (Cousin 2002). Between 1851 and 1855 Bernard also studied vasomotor activity (Olmsted and Olmsted 1952).

## Serendipity and catching an Idea

The fact that the liver formed sugar (glucose) raised new questions. How? And from what? It took another seven years before Bernard reported that “a glucose forming material existed in liver” (Bernard 1855b). Several other scientists had confirmed that the liver produced glucose, and it was generally believed that proteins were the precursors. During this period, Bernard reflected on his research strategy and investigated, among other questions, the effect of different diets and fasting on the glucose content in liver (Bernard 1865).

Bernard also started to perfuse livers with cold water to test whether sugar disappeared. In these experiments, Bernard found that all glucose had disappeared after the liver had been perfused for 40 min with cold tap water. Importantly, Bernard always measured the sugar content in the liver in duplicate, and one day he only finished one measurement and left the second until the next day. Bernard did not find any sugar in the sample analysed immediately after the perfusion, and he was very surprised to find much sugar in the same liver when he analysed it the next day. Bernard’s reflections on this surprising result: “*I did not know how to account for this singular variation*,* got with the same liver and the same method of analysis. What was to be done? Should I consider two such discordant determinations as an unsuccessful experiment and take no account of them? Should I take the mean between these experiments? More than one experimenter might have chosen this expedient to get out of an awkward situation. But I disprove of this kind of action ………. Ater assuring myself that there was no mistake connected with the method of analysis*,* after noting that all parts of the liver were practically equally rich in sugar*,* there remained to be studied only the elapsed time between the animal’s death and the time of my second determination. …. In physiology*,* questions of time are always very important because organic matter passes through numerous and incessant changes. Some chemical change might therefore have taken place in the liver tissue.” (*Bernard 1865*)*; p. 166.

Again, Bernard believed in his data. He believed that a chemical change might have taken place, which was responsible for the formation of sugar. Bernard now systematically investigated the reappearance of sugar in livers that had been perfused with cold water for 40 min to remove all glucose (Holmes 1974). After such perfusion no sugar was present in the liver, but after some hours at room temperature, or on the next day, the liver was again rich in sugar. These experiments were apparently quite consistent, and much sugar was formed.

Bernard described the glucogenic function of the liver in a lecture entitled “On the mechanism of the formation of sugar in the liver”, presented at the Academy of Science on September 24, 1855 (Bernard 1855b). In the original presentation and paper, Bernard did not report anything about serendipity (Bernard 1855b). In his introduction, Bernard refuted the critique of his work presented by Figuier (who had correctly found glucose in the portal vein). Bernard did not even mention Figuier by name and only referred to his reports in Comptes Rendus by volumes and pages. Next, Bernard described the support for the glycogenic function of the liver provided by Schmidt, Lehmann and Frerich (Bernard 1855b).

Bernard then carefully described an experiment in which a liver from a healthy dog, fed only meat for several days, was perfused with tap water for 40 min, which made the liver pale. Bernard boiled part of the liver and the decoctum did not contain sugar (no sign of reduction of copper (Barreswil’s reagent) or any trace of fermentation with brewer’s yeast) (Bernard 1855b). The other part of the liver was left at room temperature for 24 h. He found the liquid strongly sweet and documented the presence of sugar with brewer’s yeast fermentation (Bernard 1855b). Bernard concluded that (1) the liver contained sugar, which was soluble in water and removed by perfusion, and (2) the liver contained some other material, which could form glucose.

Next, Bernard reported that the sugar-forming material was completely insoluble in alcohol. In this experiment, a liver was crushed and sieved to remove the vessels and nerves, and the pulp was collected for further studies. The liver paste was then stirred, macerated and washed with cold alcohol to remove sugar and the pulp was collected on a filter, placed on a Joseph paper and dried in an oven at a temperature below 40 °C. Importantly, when the dried liver powder was added to water, sugar was formed after a few hours. To confirm that sugar was not already present in the liver powder, a sample was boiled for a few minutes, which completely prevented the formation of sugar. Bernard concluded that sugar formation was heat sensitive, indicating the involvement of protein.

At the end of the paper Bernard pointed out that “*during life this matter is constantly renewed in the liver tissue under the influence of nutrition; is constantly transformed into sweet matter*,* which replaces in the liver the sugar that the blood current continually carries through the hepatic veins. After death*,* in a liver removed from the body*,* this material*,* under the influence of humidity*,* can continue to change into sugar until it is exhausted*.” (Bernard 1855b), p. 468). Furthermore, Bernard concluded that “*we must seek to isolate this singular hepatic material which pre-exists*,* to know how it is secreted in the liver*,* and how it then undergoes the successive transformations which change it into sugar*” (Bernard 1855b), p 469).

This description of the physiological function of liver glycogen is impressive, considering the data/facts Bernard had available, and portray him at his best.

## Isolation of glycogen and comments to the 1857 paper

Bernard reported the existence of glycogen to the Société de Biologie on Saturday March 21, 1857 (Young 1957). On March 23, Bernard presented his results to the Academy of Science and submitted the paper entitled “Sur le mécanisme physiologique de la formation du sucre dans le foie”, which was published in Comptes Rendus (Bernard 1857c). The paper was translated to English shortly after and occurred in The Biochemist: A monthly journal of physical and chemical science as “On the physiological mechanisms of the formation of sugar in the liver” published in two parts (Bernard 1857a, b). Since the manuscript is more than 100 years old, the copyright has expired, and the translated version with comments is included (Supplementary materials).

Starch had been known since ancient times (Seetharaman and Bertoft 2012) and it had been shown that iodine stained starch in the early 19th century (Pesek and Silaghi-Dumitrescu 2024). Gottlieb Kirchhoff (1764–1833) had reported that starch was degraded to glucose by boiling it in dilute sulfuric acid in 1811 (Kirchhoff 1811; Völksen 1949). Moreover, Payen and Persoz had described a method to isolate diastase (amylase) in 1833 and shown that the enzyme rapidly degraded starch to glucose (Payen and Persoz 1833). Bernard used these methods to document that the “glucogenic matter” isolated from livers had similar properties to starch.

We will briefly summarise the key findings in Bernard’s article describing the isolation and properties of glycogen (Bernard 1857c).

The background for the study was that Bernard had shown that the liver contained “peculiar matter” that could form sugar in 1855 (Bernard 1855b). Bernard had also shown that the material was insoluble in alcohol and that cooking destroyed the ability of the liver to form sugar. Initially, Bernard had thought that the sugar-forming material was protein, but he realised that it was the ferment (enzyme) producing glucose that was destroyed by cooking. Bernard now used this creativity and experimental experience to isolate glycogen.

Bernard described the method for purifying glycogen from a dog fed meat only. The liver, still warm, was cut into very thin slices and dropped into boiling water to coagulate the liver and stop glycogenolysis. The coagulated liver slices were ground in a mortar and boiled for 45 min in sufficient water to cover the tissue and still obtain a concentrated extract. The cooked liver was then pressed in a cloth and filtered. The fluid had an opaline tint. To this liquid, 4–5 volumes of 38–40 º alcohol were added, which formed an abundant flocculent precipitate (yellow or milky white) that contained the glucogenic matter and proteins. The precipitate was washed several times with alcohol and dried.

This precipitate (crude glucogenic matter) was described as granular, greyish, and sometimes gummy. The crude glucogenic matter redissolved in water with an opaline tint and was entirely precipitable with alcohol. This solution was coloured by iodine but contained no sugar (did not reduce copper sulphate or ferment with brewer’s yeast).

To purify the crude glucogenic matter further, it was boiled with very concentrated potassium hydroxide (KOH) for 15–30 min, which degrades/hydrolyses proteins. The solution was filtered and 4–5 volumes of 38–40 º alcohol were added and stirred with a glass rod to precipitate the glycogen. The precipitate was washed several times in large quantities of alcohol. The glycogen pellet was dissolved in water, acetic acid was added to saturate the potassium carbonate, and the mixture was then heated with alcohol, which precipitated the “glucogenic matter” (glycogen).

The glucogenic matter (glycogen) then lost its granular form and became a white, very fine flocculent substance that resembled flour when dried. The prepared matter had properties similar to hydrated starch (having no odour or taste and being soluble in water). Iodine produced a colouration, and the matter did not contain nitrogen. The material also did not reduce copper sulphate or undergo alcoholic fermentation with yeast, and was completely insoluble in alcohol.

The hepatic glucogenic matter had characteristics that rendered it in every respect analogous to hydrated starch. The glucogenic matter was neutral, had no odour or taste, and resembled starch on the tongue. It dissolved, or more correctly, was suspended, in water, to which it gave a strongly opaline tint. Microscopic examination showed no peculiarity of structure.

The next step was to show that the glucogenic matter was a carbohydrate, analogous to starch, and could be decomposed into sugar. To do that Bernard boiled the glucogenic matter in mineral acid or added diastase or saliva, which were methods known to degrade starch to glucose. Bernard found that these methods also transformed the “glucogenic matter” to sugar, documented by reduction of Barreswil’s solution and fermentation with yeast. Bernard reported that diastase transformed the “glucogenic matter” into sugar in a few minutes at body temperature. Moreover, the solution was not stained by iodine when sugar had been produced, supporting the theory that the glucogenic matter, analogous to starch, had been degraded to sugar.

Torrefaction (the process of thermally degrading organic material in nitrogen or an inert environment within a narrow temperature range of 200–300 ℃) produced the same changes to “glucogenic matter” as to starch. Bernard also supplied a sample of the solution to the physicist Jean-Baptiste M. Biot, who showed that it deflected polarised light to the right as expected (as starch does). Bernard concluded that the glucogenic matter had analogous properties to vegetable starch.

In the second part of the article (part II in The Chemist), Bernard discussed the physiological implications of his findings. Today these ideas are mostly of historical interest. Bernard had shown that the glycogen isolated from livers in all respects was comparable with starch in plants and discussed the physiological function of glycogen. He compared the synthesis and degradation of starch in seeds with the glucogenic function of the liver, and reflected on the regulatory role of the circulatory and nervous systems in the synthesis and degradation of the glucogenic matter in the liver. Bernard’s comparison of the synthesis of carbohydrates in animal (glycogen) and plants (starch) was important in his arguments for the significance of his findings. Bernard’s findings are often considered the final blow to Dumas’ theory that only plants synthesised carbohydrate proteins and fat, and animals merely degraded energy substrates. Bernard’s findings established the modern view of metabolism in animals, in which the dynamic synthesis and degradation of glycogen and other macromolecules are central.

In the second part, Bernard explained that glycogen was degraded to sugar in livers removed from animals, and considered this a physio-chemical reaction independent of life. Bernard wrote: “*It is evident from what I have previously said*,* that the glucogenic matter created by the liver in the physiological state during life*,* is capable of changing into sugar*,* simply by means of a ferment*,* and independently of the vital influence*” (Bernard 1857a). However, Bernard still believed that a vital force was required for the synthesis of glycogen and wrote later: “*The first action is entirely vital*,* so called because its accomplishment does not take place outside the influence of life*,* consisting in the creation of glycogen matter in the living hepatic tissue*” and that “*The glucogenic matter must be created by the vital activity of the organ;*” (Bernard 1857b).

Bernard was a determinist and fought the vitalism that interfered with his physio-chemical theories, but he accepted certain forms of vitalism (Kanamori 2005). Importantly, Bernard aimed to establish physiology independently from both anatomy and chemistry, and criticised Mialhe’s research for being founded too much in chemistry (Holmes 1974). A complete acceptance of a pure chemico-physical foundation of life might have weakened Bernard’s arguments and his ambition to establish physiology as an independent area. Bernard noted his reflections in Cahier Rouge (written between 1850 and 1860) and speculated much about the role of vitalism and materialism for the physiological properties^3^ (Bernard 1967). Bernard wrote for example: *Relation of the chemical and physiological effects: The physio chemical phenomena do not produce the physiological properties*,* and it can be admitted that the physiological property can be converted into physio chemical phenomena but not the reverse* (Bernard 1967); p.78. However, Bernard thought much about life and creation and wrote in 1865 in An Introduction to the Study of Experimental Medicine: “*To-day we differentiate three kinds of properties exhibited in the phenomena of living beings: physical properties*,* chemical properties and vital properties. But the term “vital properties” is itself only provisional; because we call properties vital which we have not yet been able to reduce to physico-chemical terms; but in that we shall doubtless succeed some day*.” Therefore, Bernard might have applied “vital force” in the paper about isolation of glycogen (Bernard 1857c) simply to describe that the mechanisms for glycogen synthesis remained unknown.

Importantly, 20 years after Bernard isolated glycogen, he performed experiments on yeast fermentation, but died early in 1878 before the results were published. Bernard’s results were published after his death by Marcellin Berthelot (1827–1907), who reported that «*alcoholic fermentation was the result of the action of molecules then called “soluble ferments”*,* and today called “enzymes”»* (Habert 2022). Habert interprets this to mean that Bernard explained fermentation as a pure chemical process, which strongly contradicted Pasteur’s theory (Habert 2022). Bernard never questioned the chemico-physical foundation for life, but he did not completely refute vitalism publicly.

**Fig. 1:**
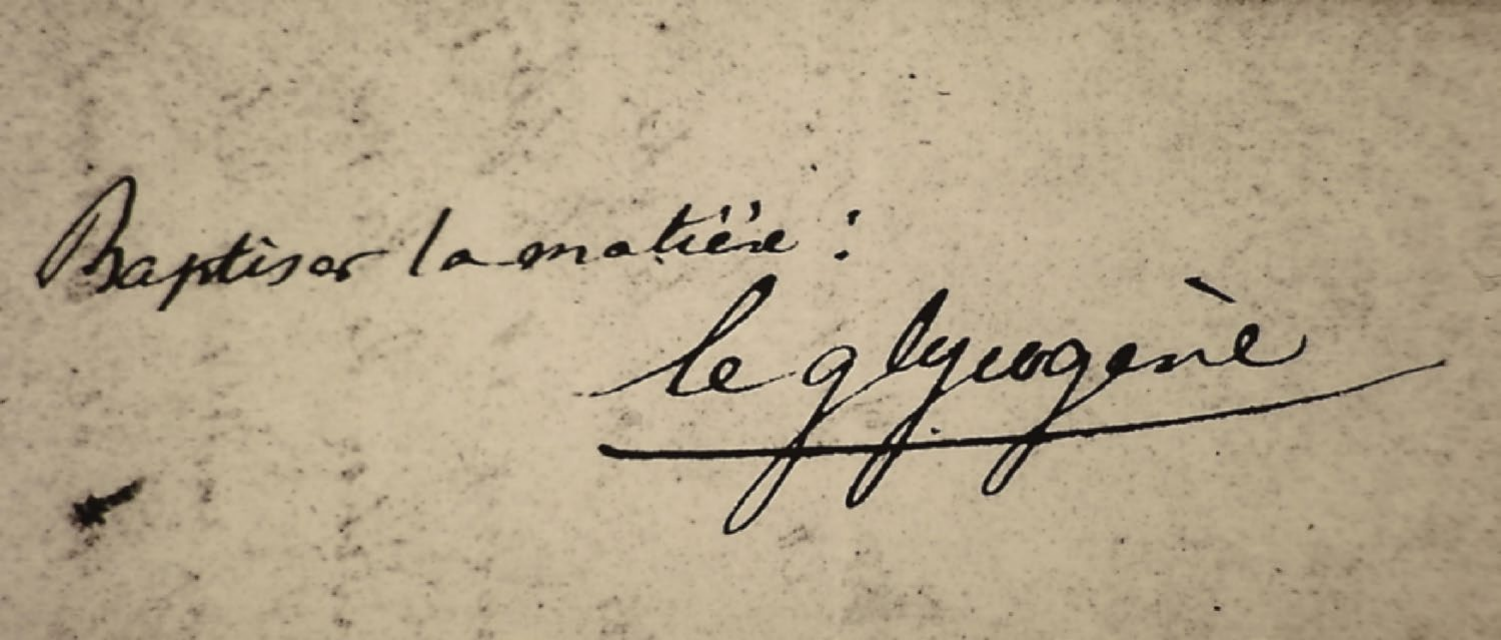
Bernard baptised the glucogenic matter for “*le glycogène*”. The exact date of Bernard’s handwritten note is not known. The photo was taken between 1980 and 2000 commissioned by a former museum curator: Ms. Jacqueline Sonolet, or Ms. Annick Opinel. Développement Atelier Dubure - Paris 5ème. The sentence, in Claude Bernard’s handwriting, is reproduced exactly on a wall in the Claude Bernard Museum. We thank Collège de France and Musée Claude Bernard for permission to use the photo. The Claude Bernard Museum holds the copyright of the photo

Bernard baptised the glucogenic matter for “*le glycogène*” which means “sugar-producer”, derived from the Greek word glyco- (sweet) and the French -gène (producer) (Fig. 1). The exact date of Bernard’s handwritten note is not known (Bernard 1952), but he used the word in a short communication in Compte Rendu in 1855 (Bernard 1855a). Bernard may have been inspired by Antoine Lavoisier who had named oxygen (“*oxygène”*) from the Greek roots oxys (acid) and -genēs (producer) in 1777. Although it turned out to be wrong, Lavoisier had several reasons for believing that oxygen was “acid-producer” and the word has survived (Crosland 1973; Holmes 1985). Bernard used term “*la matière glycogène*” in the paper on the isolation of glycogen (Bernard 1857c), which was translated to “glucogenic matter” in the English translation (Bernard 1857a). The first use of the word glycogen in English is dated to circa 1864 according to the Merriam-Webster Dictionary.

## Bernard was not always right and was a bit lucky

Not all Bernard’s data on glucose metabolism were correct, but the glucogenic function of the liver still stands. Bernard was an excellent physiologist and his dexterity in vivisection was outstanding and important in his success. Holmes described Bernard’s knowledge of chemistry as limited, and the copper method for the measurement of glucose was not particular accurate in Bernard’s hands (Holmes 1974).

The fact that Bernard did not find glucose in the portal vein is of course wrong, but Bernard’s analyses were not very sensitive. In 1855, Figuier correctly found sugar in the portal vein and was quite determined in his criticism of Bernard’s work (Chevallier 2024). The Academia of Science established a committee to clarify the controversy. The members of the committee were Dumas, Pelouze and Rayer, and they supported Bernard’s view because Figuier only could document sugar using the copper method, not with yeast fermentation (Olmsted and Olmsted 1952); p. 201. The matter was discussed in detail by Chevallier in the article “Claude Bernard and sugar: the controversy with Figuier and Bérard” (Chevallier 2024).

In 1856, Chauveau developed more accurate methods for the measurement of glucose and demonstrated that glucose was always present in blood (Chauveau 1856; Young 1937). However, this was not generally accepted until it was confirmed by Bernard (Young 1937). In “*Leçons sur le diabète*” published in 1877, Bernard still concluded that “*My earlier results were valid for the conditions under which they had been obtained; they are now merely refined by the application of more precise conditions*” (Young 1937); p. 55.

The methodology for the collection of blood was quite different from the techniques used today. Bernard normally killed the animals before he collected blood, and often conducted other investigations before the blood was collected. The methods Bernard used to kill the animals were important to his ability to find glucose in the liver and the portal vein. Both a blow to the skull and sectioning of the medulla oblongata activate the sympathetic nervous system and stimulate glucose production. Without such activation Bernard would never have found sugar in the blood (Grmek 1968). Today we know that glucose release from the liver is under sympathetic regulation (Shimazu and Fukuda 1965; Zsombok et al. 2024) and biochemical textbooks describe adrenaline-stimulated glycogenolysis in the liver (Newsholme and Leech 1983). Moreover, adrenaline infusion or injection increases blood glucose (Jensen et al. 2011a; Kolnes et al. 2015). Pavy understood that the treatment of the animals before sampling blood influenced the results and that “excitement” increased glucose concentration, which led him to criticise Bernard’s work (Pavy 1860; Young 1937). Pavy also criticised Bernard for not waiting long enough after boiling samples with Barreswil’s reagent before the colour was read (Young 1937).

Grmek wrote *“How lucky he was to ignore some facts. First of all*,* there is in every case some amount of sugar in the blood of the portal vein. It was only by a special property of his chemical test that a gradual difference was transformed into an all-or-none reaction. The large amount of sugar in the blood of Bernard’s dogs resulted from the manner of killing (section of the medulla oblongata) and should be interpreted as an exceptional*,* pathological condition. It is astonishing “how much instinctive judgment and even sheer luck contributed to a discovery which Bernard*,* with a good deal of justification*,* believed to be based upon the strictest experimental proof.” And how interesting it is to measure the extent to which a great scientist reconstructs his own previous thoughts to fit his later point of view”* (Grmek 1968).

## Research on glycogen after Bernard

Later in 1857, A. Sanson reported that glycogen also existed in muscle and some other tissues (Sanson 1857) and glycogen metabolism rapidly became a key issue in physiology after Bernard isolated and described it in the liver (Pflüger 1905). More than 150 years after Bernard described glycogen, the topic still holds major scientific interest and many questions remain unresolved (Jensen and Kolnes 2024; Jensen et al. 2011b). Although the glycogen concentration is much higher in the liver than in skeletal muscles, the total carbohydrate storage in humans is 4–5 times higher in muscles than in the liver (Jensen et al. 2011). Bernard did not understand the function of muscle glycogen (Young 1957), but muscle glycogen is an important energy substrate during exercise, and depletion of muscle glycogen causes fatigue (Bergström et al. 1967; Jensen and Kolnes 2024). We recently reported that 50% of the glycogen content in skeletal muscle remained after 7 days’ fasting (water only) in healthy humans (Kolnes et al. 2025). This finding suggests that muscle glycogen has a key role in survival by maintaining anaerobic capacity, which is important for fight-or-flight activities (Kolnes et al. 2025). The work on glycogen and fasting that Bernard initiated almost 180 years ago is still active and ongoing.

The basic principles Bernard used to isolate glycogen are still used. Glycogen is still precipitated with alcohol, although we now use a higher alcohol concentration (final concentration 66%) compared to Bernard’s method (Franch et al. 1999). We still use KOH and acetic acid as Bernard did, but we start the analyses by dissolving muscle tissue in heated KOH (70 °C for 20 min) before neutralising with acetic acid and finally precipitating with ethanol (Franch et al. 1999; Lai et al. 2007). In combination with ^14^C-labelled glucose, this is a powerful and robust method for the measurement of glycogen synthesis (Franch et al. 1999).

## The legacy of Claude Bernard

Bernard’s legacy is unquestionable. However, although his physiological contributions were significant, much his original work is not often cited. Bernard’s work was part of the beginning of modern physiology, and he fought to establish experimental physiology as an independent discipline. His teaching attracted many international students/physicians, and this may have been as important as his own work for the progress in physiology that was made in the 50 years after his death.

For many physiologists, Bernard is best known for introducing the concept of homeostasis. Bernard introduced the concept of “The constancy of the internal environment” (*La fixité du milieu intérieur)* in “Lectures on the phenomena of life common to animals and plants” (*Leçons sur les phénomènes de la vie communs aux animaux et aux végétaux in the section*) in the section titled “Constant or free life” (*Vie constante ou libre*) (Bernard 1878a). We quote from the English translation from 1974: “*I believe I was the first to insist upon this idea that there are really two environments for the animal*,* an external environment in which the organism is placed*,* and an internal environment in which the elements of the tissue live (*Bernard 1974*)*; p. 83. Bernard explained next that “*The constancy of the internal environment is the condition for free and independent life; the mechanism that make it possible is that which assured the maintenance*,* within the internal environment*,* of all the conditions necessary for the life of the elements*” (Bernard 1974), p. 84). Walter Cannon (1871–1945) introduced the term homeostasis in 1926 and described the phenomenon carefully in 1929 in his article “Organization for physiological homeostasis” (Cannon 1929). Cannon’s teacher and supervisor was Henry Pickering Bowditch who studied in Paris with Claude Bernard in 1868–1869 (Cannon 1922). Moreover, Cannon visited Paris in 1918 (Cooper 2008) and may have been inspired by working in the same location as Claude Bernard.

Bernard’s legacy is also manifested in his famous book “An Introduction to the Study of Experimental Medicine”. Bernard was already a highly regarded scientist when he presented the book in 1865, and it immediately made a tremendous impression. Louis Pasteur (1822–1895) wrote: “*Nothing so complete*,* so profound*,* so enlightening has never been written on the true principles of the difficult art of experimentation. This book will exert an immense influence on medical science*,* its teaching*,* its progress and even its language*.” (Tarshis 1968); p. 110.

Bernard has much to teach modern students, and “An Introduction to the Study of Experimental Medicine” should be required reading in all courses on the theory of science for natural sciences. The book may be more relevant now than ever. Bernard stated: “*When we propound a general theory in our sciences*,* we are sure only that*,* literally speaking*,* all such theories are false. They are only partial and provisional truths which are necessary to us*,* as steps on which we rest*,* so as to go on with investigation*.” Bernard preceded Popper by nearly 100 years in seeing the importance of falsification (Recio 2004). More importantly, Bernard looked at falsification through the eyes of a scientist.

Currently, irreproducibility in research is a topic of concern (Flier 2022). As this paper illustrates, Bernard and other scientists in the 19th century had also problems obtaining reproducible data. Bernard’s view was that “*We must never make experiments to confirm our idea*,* but simply to control them*,* which means*,* in other terms*,* that we must accept the results of experiments as they come*,* with all their unexpectedness and irregularity*” (Bernard 1865). Importantly, Bernard challenged the existing theories and his ability to build new theories around the facts he collected was extraordinary.

## Supplementary Information

Below is the link to the electronic supplementary material.

Supplementary Material 1

## Footnotes

1 The eruption of Mount Tambora, Indonesia, in April 1815 was the largest outbreak in more than 1000 years and caused the coldest summer temperature in Europe in 1816 recorded, which resulted in crop failures and major food shortages. France was severely affected.

2 The method builds on the reducing property of glucose converting Cu^2+^ (blue) to CuO_2_, which has a red colour (Cu_2_ [blue] + aldehyde → CuO_2_ [red] + carboxylic acid). In this paper we will also use “Barreswil’s reagent” to describe the method for determination of glucose. Reduction of Barreswil’s reagent indicated that sugar was present.

3 Bernard wrote in Cahier rouge: *Relation of the chemical and physiological effects.* *The physio chemical phenomena do not produce the physiological properties*,* and it can be admitted that the physiological property can be converted into physio chemical phenomena but not the reverse.* Page 78 in Bernard (1967). The Cahier Rouge of Claude Bernard. Translated by H.H Hoff, L Guillemin, and R. Gullemin. Schenkman Publishing Company, Inc.
