# Supplementary material for: Claude Bernard’s route to the isolation of glycogen: the journey that changed scientific views on the physiological role of the liver and animal metabolism
Source: Eur J Appl Physiol. 2025 Dec 19;126(2):629–47. doi: 10.1007/s00421-025-06080-x (PMC12948771; doi:10.1007/s00421-025-06080-x)
Supplement: Supplementary file 1 — Supplementary Material 1 [file 421_2025_6080_MOESM1_ESM.pdf]

The Chemist published a translated version of Claude Bernard famous paper: «*Sur le mécanisme physiologique de la formation du sucre dans le foie*» in 1857. The English translation of held the title: «On the Physiological Mechanism of the Formation of Sugar in the Liver».

The paper is commented by Jørgen Jensen, Department of Physical Performance, Norwegian School of Sport Sciences (2025).

THE CHEMIST: A MONTHLY JOURNAL OF CHEMICAL AND PHYSICAL SCIENCE.
EDITED BY JOHN AND CHARLES WATT.
LONDON: ALEXANDER WATT, 8.3, DEAN STREET, SOHO PIPERS, PATERNOSTER ROW; AND ALL BOOKSELLERS.

The first half of Bernard's paper was published in The Chemist: a Monthly Journal of Chemical and Physical Science, 1857, vol. 4 (New Series), p. 553-557.

PHYSIOLOGICAL & PATHOLOGICAL CHEMISTRY.

On the PHYSIOLOGICAL MECHANISM of the FORMATION of SUGAR in the LIVER. By M. CLAUDE BERNARD.

In a former communication[1] I have shown results which appeared to me incompatible with the various chemical theories previously promulgated to explain this singular production of saccharine[2] matter in an animal organism[3]. These experiments caused me to examine the mechanism of the glucogenic function of the liver[4] from a new point of view, and they have led me to think, contrary to those previous opinions, that the sugar was not formed at once in the hepatic tissue by the direct decomposition of such or such an element of the blood, but that it is always preceded by the creation of a peculiar matter capable of forming it by a sort of secondary fermentation[5]. I concluded that to make any fresh progress in the glucogenic question, it would be absolutely necessary to isolate this hepatic material existing before the sugar, so as to study its characters and to determine the physiologico-chemical[6] role which it plays.

All the views which I delivered from my first work have been fully proved by experiment, and I have now to communicate the positive existence and isolation of the glucogenic matter

[1] In the original manuscript in French, Claude Bernard referred to his publication from 1855 where the formation of sugar in the liver was described. Bernard, C. (1855). Sur le mécanisme de la formation du sucre dans le foie. Comptes Rendus Acad d Sci, 41, 9. (461-469)
[2] Saccharine: sugar, having the qualities of sugar.
[3] Dumas and Boussingault promoted the idea that only plants synthesised sugar, fat and proteins. Animal only degraded the nutrients. See Dumas, J. B. (1841). XLVII. On the chemical statics of organised beings. Philosophical Magazine Series 3, 19, 11
[4] Bernard reported that the liver produced sugar (glucogenic function) in 1848.
[5] Ferment was used to describe the molecule that catalysed a degradation (comparable to enzyme). By secondary fermentation Bernard suggested that the common fermentation was not sufficient to describe what happened. In 1857, the mechanisms for degradation of nutrients were basically unknown and only few enzymes catalysing degradation were described.
[6] In the late 19th century "physiological chemistry" described what today is biochemistry.

existing before the sugar, to which it will become very easy, as will be seen, to assign its role in the physiological mechanism of the formation of sugar in the liver.

It is evident from what I have previously said, that the glucogenic matter created by the liver in the physiological state during life, is capable of changing into sugar, simply by means of a ferment[7], and independently of the vital influence[8]. The experiment of the washed liver which again filled itself with sugar is the proof of this.

The entire difficulty remaining was, consequently, to separate the matter in question from the tissue of the liver, and to isolate it from the ferment which accompanies it. I shall not enter into details of all the means by which I have attained this result, because the knowledge of all those difficulties becomes useless and even disagreeable to the mind when the question is explained and simplified. I will only say that when I saw that cooking stopped the formation of a fresh quantity of sugar in the washed liver, I for some time falsely imagined that the glucogenic matter must be an albuminoïd[9] material, alterable by heat, whereas in reality it was only that the ferment[10] was destroyed by cooking; I ascertained that this was the case by causing the washed and cooked liver to ferment by means of the ferment removed from fresh hepatic tissue.

Hence it was proved to me that the hepatic glucogenic matter had the faculty of dissolving in boiling water, and that it might thus be separated from its ferment, which remained coagulated with the other albuminoïd matters of the liver. The process is thus fully traced. It is true that these things might be differently interpreted by considering that the glucogenic matter extracted by boiling, or even cold water, is not the primitive matter itself, but already the result of a transformation. This interpretation, which appears to me less probable, would, however, in no respect alter the general signification of my experiments in a physiological point of view.

It is probable that a great many other means of extracting the glucogenic matter from the liver may be considered more advantageous: I shall simply give the process which I preferred.

The liver is taken still hot and bleeding from a healthy animal; any animal will do, and fed on any kind of food. But, to simplify the question on this point, I will observe that the following experiments were made on dogs, fed exclusively on meat. The tissue of the liver is cut into very thin slices, which are immediately thrown into water which is kept constantly boiling, so that the tissue of the organ is suddenly coagulated, and that the glucogenic matter in contact with its ferment may not have time to change into sugar under the influence of a too slowly rising temperature. The pieces of coagulated liver are then brayed in a mortar, then this species of bouillie[11] of liver is allowed to cook for three - quarters of an hour or an hour, in sufficient water only to cover the tissue, so as to obtain in the concentrated extraction as large a quantity as possible of the matter which is capable of changing into sugar. The tissue of the liver thus cooked is then pressed in a cloth, or under a press, and the liquid extract is poured on a filter, through which it passes with an opaline tint. To this liquid is then added four or

[7] Ferment was used to describe what we today call enzyme.
[8] In 1857 the theory about a vital force determining life was still dominant. Bernard also believed that life (vital action) was necessary for synthesis, but recognised that sugar production from glycogen was chemical.
[9] Resembling albumin. Protein
[10] Enzyme. The word enzyme was coined by Wilhelm Kühne in 1878.
[11] Porridge or mash.

five times its volume of alcohol at 38° or 40°, and under its influence we see formed an abundant flocculent precipitate of a yellowish or milky white, which is formed of the glucogenic matter itself, still retaining sugar, bile, and other undetermined nitrogenous products. All the precipitate, again collected in a filter, is then washed several times with alcohol, so as to deprive it as far as possible of the sugar and soluble biliary matters. In this state the dried precipitate is a greyish substance, sometimes gummy, to which has been given the name of crude glucogenic matter. It possesses the property of redissolving in water, to which it always communicates a strongly opaline tint, and from which it is entirely precipitable by concentrated alcohol.

The aqueous solution of this crude glucogenic matter, before being treated with potassium hydroxid, is coloured by iodine[12], does not reduce the salts of copper dissolved in potassa[13], and does not ferment with yeast. Nevertheless, if left for a long time, this substance appears to me to become partially changed into sugar; this is doubtless when some foreign matters are still mixed with it.

To purify this glucogenic matter[14], and free it from nitrogenous matters, such as the very slight traces of glucose which it may still retain, it is boiled in a solution of very concentrated caustic potassa[15] for a quarter or half-an-hour, an operation which makes no change in it, and does not alter its fundamental properties; it is then filtered with the addition of a little water, and the whole solution is again precipitated by the addition of four or five times its volume of alcohol at 38° or 40°. Then, stirring it with a glass rod, the precipitate divides, having at first had a great inclination to adhere to the vessel. After repeated washings with large quantities of alcohol, the potassa is removed as far as possible, and the glucogenic matter is found under the form of a granular substance, almost pulverulent. This matter, thus prepared, still retains a certain quantity of potassa, which cannot be removed by simple washings with alcohol; for this purpose the matter must be redissolved in water, the carbonate of potassa saturated with acetic acid, and then heated again with alcohol, which precipitates the matter, and separates it from the accetate of potassa, which remains soluble in the liquor. The glucogenic matter then loses its granular form, and assumes the aspect of a white, very fine floculent substance, when in suspension in the alcohol; pulverulent and resembling flour when dried.

Thus prepared, the glucogenic hepatic matter possesses characters which render it in every respect analogous to hydrated starch which has begun to decompose. This is a neutral matter, without odor or taste, resembling starch on the tongue. It dissolves, or more correctly, is suspended, in water, to which it communicates a strongly opaline tint. Microscopical examination shows no peculiarity of structure.

---

[12] Iodine was known to stain for starch, which is composed by glucose molecules. The fact that iodine stained "glucogenic matter" indicated that it was composed of glucose molecules similar starch, which is correct.

[13] The method Bernard used to measure sugar. The method builds on the reducing property of glucose which concerts Cu^{2+} (blue) to CuO_2 in alkaline solutions, which has a red colour. Barreswil developed the method for determination of glucose.

[14] Bernard wrote «matière glycogène».

[15] Caustic potassa: Potassium hydroxide; KOH.

Iodine gives a coloration which varies in intensity from deep violet to a pale maroon red; the coloration is rarely purely blue. When heated to redness with soda-lime, this hepatic matter does not disengage ammonia, which indicates that it contains no nitrogen[16]. (The crude glucogenic matter treated in the same manner does disengage ammoniacal vapors.) It does not reduce the salts of copper dissolved in potassa[17], does not undergo alcoholic fermentation under the influence of yeast[18], is completely insoluble in strong alcohol, and precipitable from its aqueous solution by the sub-acetate of lead, animal charcoal[19], &c. *

But the property of the hepatic matter which interests us the most is that which relates to its transformation into sugar. It is then that the physiological analogies between this substance and hydrated starch are seen most clearly. We see that all the influences, without a single exception, which transform vegetable starch into dextrine and glucose, will likewise change the glucogenic matter of the liver into sugar, passing through an intermediate stage analogous to dextrine. Thus, prolonged boiling with the mineral acids[20] diluted with water, the action of vegetable diastase[21] and that of all the analogous animal ferments, such as the pancreatic juice[22] or tissue, the saliva[23], the blood, & c., very quickly transform the glucogenic matter into sugar. When this gradual transformation takes place, the glucogenic solution, from being opaline, becomes by degrees transparent, and at the same time it loses the faculty of being colored with iodine. But soon afterwards, and only when the definitive change into sugar has been effected, the solution acquires the properties of reducing the salts of copper dissolved in potassa, of fermenting under the influence of yeast, and giving alcohol and carbonic acid[24]. I must add that the action of the diastasic ferments[25] produces this transformation into sugar in a few minutes, when care is taken to keep the liquids at about the natural heat of the body, between 35° and 45° C. (95° and 113° F.) The aqueous solution of the hepatic glucogenic matter does not change spontaneously into sugar; it changes only with great difficulty when left to itself, and partially resists the putrefaction of the cooked tissue of the liver.

** When the tissue of the fresh liver is brayed, and the hepatic pulp coagulated cold, with a sufficient quantity of alcohol at 38°or 40°, we precipitate the glucogenic matter and its ferment. After having, by repeated washings with alcohol, removed the sugar and dried the matter which is reduced to a sort of powder of tissue of liver, if we replace it in cold water we obtain an opaline solution, containing the hepatic glucogenic matter and its ferment. What proves this is, that if this solution is left to itself, it becomes very rapidly charged with sugar. When the transformation into sugar is complete, the ferment may be precipitated by alcohol separate from the sugar, and thus isolated. But when we add alcohol to the solution before the sugar appears, we precipitate the glucogenic matter with its ferment. When the matter thus obtained is boiled with caustic potassa, there is a disengagement of ammonia produced by the destruction of the nitrogenous matter of the ferment mixed with the glucogenic matter.*

---

[16] Nitrogen is present in proteins. Therefore, «no nitrogen» indicated that the material did not contain protein.
[17] Glucose was determined by its reducing property. «It does not reduce the salts of copper dissolved in potassa» therefore indicated that no sugar was present.
[18] The ability of yeast to induce fermentation. Therefore, «does not undergo alcoholic fermentation under the influence of yeast» indicated that no sugar was present.
[19] Fine charcoal prepared by calcining bones.
[20] Mineral acid was known to degrade starch to glucose.
[21] Diastase is synonym for amylase. It was known that diastase degraded starch to glucose.
[22] Pancreatic juice contains diastase, which was known to degrade starch to glucose.
[23] Saliva was known to degrade starch to glucose. Saliva contains diastase (amylase).
[24] The sentence indicates that sugar became present.
[25] Diastatic ferments: amylase.

Torrefaction[26] and the limited action of the ferments and mineral acids, change the glucogenic matter into a body which has characters in every respect similar to those of dextrine.

This substance is insoluble in concentrated alcohol; dissolves in water, giving a transparent solution; it is no longer sensibly colored by iodine; does not reduce the salts of copper dissolved in potassa; does not ferment with yeast, and deviates the plane of polarisation to the right.

In a very dilute aqueous solution of this matter examined by M. Biot, at the Collège de France, in a tube of 320 millimetres, a well characterised deviation to the right[27] was ascertained, with very marked development of tints round the minimum of intensity of the extra ordinary image.

From all the experiments which I have before described, it is consequently established that the liver of dogs fed exclusively on meat possesses the peculiar property, exclusive of every other organ of the body, of creating a glucogenic matter quite analogous to vegetable starch, and capable like that of being changed finally into sugar, passing through an intermediate stage similar to dextrine[28].

Without any doubt, the study of the glucogenic matter of the liver must not stop here. It was necessary to ascertain exactly its elementary composition and its constitution; to know whether this matter changes entirely into sugar, and whether in this transformation other products might not be formed; and to make a profound study of the very apparent parallelism between the transformation into sugar of this glucogenic matter of the liver, and the transformation into sugar of vegetable starch. This study belongs to chemists. I am at present satisfied with having proved the existence of this peculiar substance, which always precedes the appearance of sugar in the liver, and establishing a fact, which is capable of powerfully elucidating the physiological mechanism of the formation of sugar in animals, and at the same time of furnishing conclusions of the greatest interest to animal physiology.

(To be continued.)

[26] Thermally degrading organic material in a nitrogen or inert environment within a narrow temperature range of 200–300 °C.
[27] Bernard here demonstrated that glycogen deviated polarised light to the right as starch does.
[28] Glucose. Jean Baptiste Dumas termed glucose in 1838. Kekulé later suggested the term dextrose because aqueous glucose solution turned polarized light to the right (Dexter (Latin) meaning right).

Part 2.

On the PHYSIOLOGICAL MECHANISM of the FORMATION OF SUGAR in the LIVER.

By M. CLAUDE BERNARD.

The Chemist: a Monthly Journal of Chemical and Physical Science, 1857, vol. 5 (New Series), p. 620-623

The physiological formation of sugar in animals should be considered as I have already said, not as a phenomenon of direct chemical separation of the sanguineous elements at the moment of the passage of the blood through the liver, but as a function performed by the succession and combination of two essentially distinct acts.

The first action is entirely vital, so called because its accomplishment does not take place outside the influence of life, consists in the creation of glycogen matter in the living hepatic tissue[29].

The second action is chemical, and may take place without the vital influence, it consists in the transformation of the glucogenic matter into sugar with the aid of a ferment.

The reunion of these two orders of conditions is necessary for the appearance of sugar in the liver. The glucogenic matter must be created by the vital activity of the organ[30]; it is then necessary that this matter should be brought in contact with the ferment which is to convert it into sugar.

The glucogenic matter is formed like all the products of organic creation, by means of the phenomena of slow circulation which accompany the acts of nutrition. As for deciding if, amid the numerous blood vessels with which the liver is provided there be any which are peculiarly charged with this nutritive circulation and others peculiarly connected with the phenomena of the chemical transformation of the glucogenic matter, that is a physiological question upon which it is at this moment unnecessary to enter. It will suffice for us to indicate in a general manner how the contact between the glucogenic matter and its ferment may take place in the living animal.

I at first thought that the ferment was peculiar to the liver, like the glucogenic matter itself; I had even succeeded in obtaining it in an isolated state. But, seeing afterwards that the sanguineous fluid possesses the property of transforming this glucogenic matter into sugar with the greatest readiness, it became impossible to think of a localisation of the ferment[31];

---

[29] This sentence emphasises that Bernard used a vital force to explain synthesis, and he was not completely detached from vitalism. Bernard wrote: "Le premier acte entièrement vital, ainsi appelé parce que son accomplissement n'a pas lieu en dehors de l'influence' de la vie, consiste dans la création de la matière glycogène dans le tissu hépatique vivant."

[30] Bernard again used a vital force to explain synthesis. Bernard wrote: "Il faut que la matière glycogène puisse être créée par l'activité vitale de l'organe ;"

[31] Bernard found that the fluid from the blood and other tissue could degrade glycogen to glucose, which he could not explain. Today we know that most cells have a small amount of glycogen and the enzymes that degrade glycogen (glycogen phosphorylase).

that extracted from the liver coming, very probably, from the blood itself. So that if out of the organism we have several ferments which operate the transformation of the glucogenic matter into sugar, in the living animal it is only necessary to admit one represented by the blood, which moreover possesses the property of rapidly changing hydrated vegetable starch into dextrine and sugar. Without entering into the intimate mechanism of this contact and into the explanation of the physiological causes which vary its intensity, which would lead us into descriptions of microscopic anatomy and phenomena of capillary circulation which will be developed elsewhere; we shall confine ourselves to saying that the observation of physiological phenomena teach us that in the liver, parallel with this slow and nutritive circulation, there must be considered another, intermittent, variable, and whose superactivity coincides with the appearance of a larger quantity of sugar in the tissue of the organ.

In digesting animals the circulation in the vena porta is super-excited, and then the transformation of the glucogenic substance is much more active, although the formation of this matter does not appear to correspond with it at that moment. This circulatory super activity may likewise be awakened without digestion; and then the same phenomena of transformation of the matter and appearance of the sugar take place. Among hibernating or benumbed animals, such as frogs for instance, the slackening of the circulation, which is connected with a lowering of the temperature, causes a diminution, and sometimes the almost entire disappearance of the sugar from the liver. But the glucogenic matter is there all the same, as may be proved by extracting it. It is then only necessary to put the benumbed frogs in the warmth to render their circulation active, and the sugar then soon appears in the liver. On placing the animals again in a low temperature we find that the sugar diminishes, or disappears to shew itself again when the frogs are again placed in a warmer place. I must add that these singular alternations of the appearance and disappearance of the sugar may be reproduced several times without the animals having any food, and by acting solely on the phenomena of the circulation by the intermediation of the temperature.

In warm blooded animals we can also act, by means of the nervous system, on the phenomena of the abdominal circulation, and afterwards secondarily on the transformation of the glucogenic matter in the liver. I have shown that if we cut or wound the spinal marrow in the region of the neck, below the origin of the phrenic nerves, we considerably diminish the activity of the hepatic circulation, so much so that after four or five hours there are no traces of sugar in the liver of the animal, the tissue of which still remains charged with glucogenic matter. It is remarkable that after this operation the temperature of the abdominal organs falls considerably, at the same time many other perturbations are produced of which I cannot now stop to speak.

I have likewise proved that by wounding the cerebrospinal axis in the region of the fourth ventricle, we produce exactly contrary phenomena; the abdominal circulation is very much accelerated, and consequently the renewal of the contact of the glucogenic matter with its ferment considerably extended. The transformation of the glucogenic matter moreover becomes so active, and the quantity of sugar removed by the blood becomes so considerable, that the animal, as is well known, becomes diabetic in this case, that is to say that the excess of sugar poured into the blood by the super-excited liver passes into the urine.

In these two cases the nervous system acts evidently on the purely chemical manifestation of a physiological phenomenon[32]. But when we analyse its mode of action we find that its effects are only mechanical, and are brought first to the motor organs of the capillary circulation, the effect of which has been to lessen or hinder, or else to extend and augment the contact of two substances capable by their properties, of reacting one on the other; they thus produce a chemical phenomenon which the nervous system regulates indirectly, but upon which it has no direct and primitive action. This view is not peculiar to the liver, and I shall prove further on that the chemical influences which are recognised in the nervous system in general are most commonly purely mechanical.

As for the conclusions which we can at this moment deduce, in a general physiological point of view, from the mechanism which we have indicated for the formation of sugar in the liver, it is impossible not to be struck with the similarity which exists in this respect between the glucogenic function of the liver and the production of the sugar in certain acts of the vegetable organism[33]. In a seed, for instance, which produces sugar during germination, we have likewise to consider two series of very distinct phenomena ; the first primitive, entirely vital, is constituted by the formation of starch under the influence of the life of the vegetable; the other consecutive, entirely chemical, which may take place without vegetable life, is the transformation of the starch into dextrine and sugar by the action of diastase. When a liver taken from a living animal continues for a certain time to produce sugar, it is evident that the vital phenomenon of the creation or secretion of the glucogenic matter has ceased; but the chemical phenomenon continues to be produced if the conditions of humidity and heat necessary for its accomplishment are realised[34]. In the same way, in the seed separated from the plant, the vital phenomenon of the secretion of starch has ceased with the vegetable life; but, under the influence of favorable physicochemical conditions, its transformation into dextrine and sugar by means of diastase may be produced. Finally, it is easily seen from these parallel observations, that the formation of sugar in the liver of animals passes through three series of successive transformations in every respect analogous to that of the formation of starch, dextrine and sugar in the seed of vegetables.

According to all the facts contained in this work, we may conclude that the question of the formation of sugar in animals has made an important step in advance, in consequence of the isolation of the glucogenic matter which always exists previously to the sugar in the tissue of the liver.

We still have to determine the organic form of this glucogenic matter, as well as the exact anatomical and physiological conditions of its formation in relation to the phenomena of development and the various physiological state of the liver. Some experiments which I have already commenced on this subject lead me to hope that it may be possible to go still further into the glucogenic question, and to localise the formation of the glucogenic matter in the peculiar elements of the hepatic organ.

---

[32] This sentence is interesting because Bernard linked the action of the nervous system to regulation of glycogen metabolism via purely chemical mechanism. Today we know that activation of the sympathoadrenal system stimulates the release of adrenaline and noradrenaline which stimulates glycogen breakdown. Adrenaline-mediated activation of glycogen phosphorylase is described in textbooks of biochemistry.

[33] Dumas had suggested that only plants had the ability to synthesis food stuff (carbohydrate, fat and proteins) and animals used the energy (but could not synthesise them).

[34] Bernard again stated that a vital phenomenon is responsible for synthesis.
